# Improvement in Contrast Sensitivity Function after Lacrimal Passage Intubation in Eyes with Epiphora

**DOI:** 10.3390/jcm9092761

**Published:** 2020-08-26

**Authors:** Sujin Hoshi, Kuniharu Tasaki, Takahiro Hiraoka, Tetsuro Oshika

**Affiliations:** Department of Ophthalmology, Faculty of Medicine, University of Tsukuba, Ibaraki 305-8575, Japan; k.tasaki1986@gmail.com (K.T.); thiraoka@md.tsukuba.ac.jp (T.H.); oshika@eye.ac (T.O.)

**Keywords:** contrast sensitivity function, epiphora, lacrimal passage obstruction, lacrimal passage intubation

## Abstract

This prospective case series aimed to investigate the contrast sensitivity function before and after lacrimal passage intubation (LPI) in eyes with epiphora due to lacrimal passage obstruction. We included 58 eyes of 51 patients who underwent LPI for lacrimal passage obstruction. The best-corrected visual acuity (BCVA), contrast sensitivity function, and lower tear meniscus were compared before LPI and one month after lacrimal duct stent removal. The area under the log contrast sensitivity function (AULCSF) was calculated for the analyses. Lower tear meniscus was assessed using anterior segment optical coherence tomography. The BCVA was comparable (*p* = 0.61) before and after LPI, while AULCSF increased significantly after treatment (before LPI: 1.29 ± 0.17, after LPI: 1.37 ± 0.14, *p* < 0.0001). Treatment resulted in a significant increase in contrast sensitivity at all spatial frequencies, 3–18 cycles/degree (*p* < 0.01 for 3, *p* < 0.01 for 6, *p* < 0.0005 for 12, *p* < 0.05 for 18 cycles/degree). The lower tear meniscus parameters improved significantly after treatment (*p* < 0.005); however, no correlation between the changes in the tear meniscus and those of the AULCSF was found. The contrast sensitivity significantly improved after LPI in eyes with epiphora due to lacrimal passage obstruction.

## 1. Introduction

Patients with epiphora owing to lacrimal passage obstruction often complain of physical discomfort, such as unwilling tearing, skin eczema, and vision-related discomfort. Although visual acuity is maintained, it is reported that contrast sensitivity function [1], optical quality [2], and quality of life (QoL) are compromised in eyes with epiphora owing to lacrimal passage obstruction [3,4,5].

Lacrimal passage intubation (LPI) is a well-established and effective method used for treating lacrimal passage obstruction to recanalize and recover the patency of lacrimal passage; this method has been employed as an alternative to dacryocystorhinostomy [6,7,8,9,10,11,12]. Moreover, dacryoendoscope and dacryoendoscopic techniques, which have recently been developed, have allowed for the improvement of the success rate and safety of LPI by facilitating direct visualization [13,14,15,16,17].

Although patients often experience an improvement in the quality of their vision, it is unknown whether the deteriorated contrast sensitivity function of eyes with epiphora owing to nasolacrimal passage obstruction recovers after LPI. It is important to assess the effect of LPI on contrast sensitivity, as contrast sensitivity is widely used for the clinical assessment of quality of vision in various diseases. As a fall in contrast sensitivity affects the quality of vision, this may be an indication for LPI in patients with epiphora due to lacrimal passage obstruction. This study aimed to investigate contrast sensitivity function in eyes with epiphora owing to lacrimal passage obstruction before and after LPI using different methods.

## 2. Experimental Section

This study was a single-institutional prospective case series that was approved by the Institutional Review Board of University of Tsukuba Hospital (H27-153) and adhered to the tenets of the Declaration of Helsinki. After the nature and possible consequences of the study were explained in detail, informed consent was obtained from all patients.

### 2.1. Patient Population

Patients with lacrimal passage obstruction who received LPI between November 2015 and July 2019 at the University of Tsukuba Hospital and had a distance best-corrected visual acuity (BCVA) of 20/20 or better, as determined by Snellen testing, were considered for enrollment. The inclusion criteria were the presence of symptoms of epiphora and at least one of the following dacryoendoscopic findings: nasolacrimal duct obstruction (NLDO), canalicular obstruction, or punctal obstruction. The exclusion criteria were congenital lacrimal duct obstruction, acute dacryocystitis, and a history of ocular surface surgery. Patients with cortical cataract formation in the central lens, intraocular lens only in one eye, other ocular diseases, or a history of treatment that might affect contrast sensitivity were excluded. In total, 58 eyes of 51 patients (men: 17, women: 34; mean age: 62.3 ± 9.6 years; range: 37–80 years) with lacrimal passage obstruction participated in our study. Table 1 categorizes the type of obstruction diagnosed in all our participants. Thirty-two eyes showed NLDO alone, and 26 eyes showed the involvement of proximal obstruction, i.e., punctal and/or canalicular obstruction with/without NLDO. Of 32 eyes with NLDO alone, 14 had complete NLDO and the remaining 18 had partial obstruction. Of 26 eyes with proximal obstruction, five had complete NLDO, nine had proximal NLDO, and the remaining 12 were without NLDO.

### 2.2. Surgical Technique and Postoperative Follow-Up

All surgeries were performed by three surgeons (TH, SH, KT). The surgical procedure of LPI performed in the current study was a combination of sheath-guided endoscopic probing (SEP) and sheath-guided intubation (SGI). This technique enables surgeons to perform lacrimal passage reconstruction under dacryoendoscopic guidance without blind manipulation [16,17]. The lacrimal passage anesthesia protocol involved an infratrochlear nerve block with 1% lidocaine and canalicular system irrigation with a 4% lidocaine solution, followed by the dilation of both puncta. Prior to SEP, a dacryoendoscope (LAC-06NZ-HS; MACHIDA Endoscope Co., Ltd., Chiba, Japan) was covered with a sheath that was prepared with an 18-gauge plastic cannula (SurFlash Polyurethane IV Catheters; Terumo Corporation, Tokyo, Japan). After a dacryoendoscope equipped with a sheath was inserted into the punctum, SEP was performed by widening the blocked section. The outer diameter of the dacryoendoscope was 0.9 mm (20 gauge). After the removal of the dacryoendoscope, the sheath was temporarily retained in the lacrimal passage and used as a guide for tube insertion during SGI. An 11-cm-long polyurethane Nunchaku-style lacrimal duct stent tube (LACRIFAST; KANEKA Corporation, Tokyo, Japan) was connected with the sheath. By retrieving the sheath through the nasal cavity, the surgeon was able to draw the lacrimal tube into the recanalized passage. The same steps were repeated for the other punctum using a combination of SEP and SGI. During this operation, the precise location of the lacrimal passage obstruction was recorded and utilized for diagnosing the type of obstruction in each case.

A lacrimal passage lavage with saline was performed every month postoperatively. The lacrimal duct stent tube was removed 2 to 3 months postoperatively, which is similar to the periods in previous reports [3,5,9,12]. In addition, a dacryoendoscopic investigation was performed to confirm whether the obstructed lacrimal passage was successfully recanalized.

### 2.3. Examination Protocol

Assessment of lower tear meniscus using anterior segment optical coherence tomography (OCT) and contrast sensitivity was performed preoperatively and 1 month after the removal of lacrimal duct stent tube to avoid the possible effects of intubation-associated ductal inflammation on the meniscus and visual function.

### 2.4. Assessment of Tear Meniscus

Cross-sectional images of the lower tear meniscus were captured vertically across the central cornea using swept-source anterior segment optical coherence tomography (OCT; SS-1000, CASIA; Tomey Corp., Nagoya, Japan). The OCT images were processed using in-built software. The principles, technique, and reproducibility of evaluating tear meniscus using this device have been described previously [18,19].

Lower tear meniscus height (TMH) and lower tear meniscus area (TMA) were calculated from the cross-sectional OCT images of the lower tear meniscus. The measurement was performed 4–5 s after blinking, with spontaneous eye opening.

### 2.5. Assessment of Contrast Sensitivity

The CSV-1000E chart (Vector Vision CO., Greenville, OH, USA) was used to measure contrast sensitivity function. The test was performed monocularly when the pupils of the eyes were undilated, and the testing distance was 2.5 m with best spectacle correction. Background illumination of the translucent chart was provided using a fluorescent luminance source of the instrument and was automatically calibrated to 85 cd/m^2^.

The CSV-1000E chart presents vertical sine-wave gratings at four spatial frequencies, i.e., 3, 6, 12, and 18 cycles/degree; each spatial frequency has eight different levels of contrast. Each row consists of eight pairs of circular patches and includes sine waves of a single spatial frequency. In each pair, one patch presents a grating, and the other patch is blank. The patients were asked to identify the patch with the grating, and the contrast level of the last correct response was defined as the contrast threshold in logarithmic values for each frequency [20]. From these data, the area under the log contrast sensitivity function (AULCSF) was calculated according to the method described by Applegate et al. [21]. In brief, the AULCSF was determined as the integration of the fitted third-order polynomials of the log contrast sensitivity units between the fixed limits of 0.48 (corresponding to 3 cycles/degree) and 1.26 (18 cycles/degree) on the log spatial frequency scale. This provides contrast sensitivity data as one number and makes statistical analysis easier. The AULCSF calculated by the average levels of contrast in each spatial frequency as described by the supplier (http://www.vectorvision.com/educational-resources/) is 1.24 in people aged 50–75 years.

### 2.6. Statistical Analyses

Normally distributed data before and after LPI were compared using a paired *t*-test (two-tailed test). Data that were not normally distributed were compared using the Wilcoxon signed-rank test. Analysis of the correlation between the difference in tear meniscus dimension (TMH and TMA) and the difference in quality of vision (BCVA and contrast sensitivity) before and after LPI were evaluated using Pearson’s correlation coefficient.

The AULCSF was compared between eyes with only NLDO and those with the involvement of proximal obstruction using an unpaired *t*-test (Student’s *t*-test).

The *p* values of < 0.05 were considered statistically significant for all analyses. Statistical analyses were performed using Statcel (add-in software for Microsoft Excel), version 4 (Microsoft Corp., Redmond, WA, USA).

## 3. Results

### 3.1. Comparison of Parameters before and after LPI

Table 2 shows the comparison of BCVA, AULCSF, TMH, and TMA before and after LPI. The BCVA was comparable (*p* = 0.61) before and after surgery, while AULCSF increased significantly after surgery (*p* < 0.0001). The lower tear meniscus parameters, TMH and TMA, decreased significantly after surgery (*p* < 0.005).

### 3.2. Comparison of Contrast Sensitivity at Four Specific Frequencies before and after LPI

**Figure 1:**
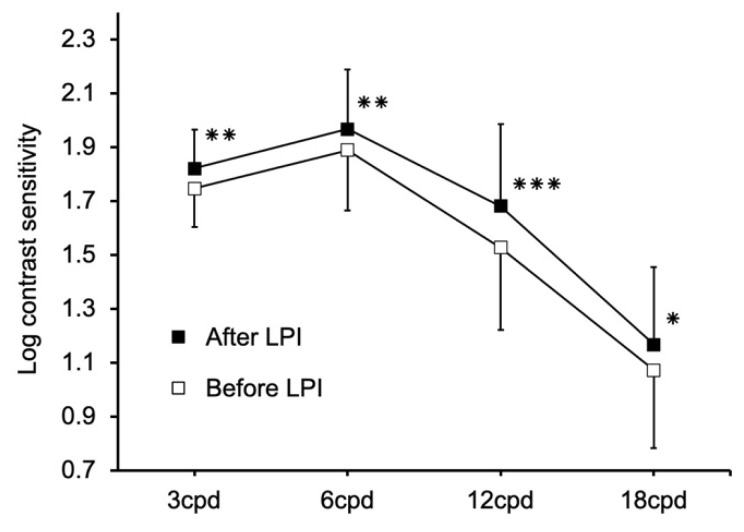
Contrast sensitivity at four specific frequencies before and after lacrimal passage obstruction. Treatment resulted in significant increases in contrast sensitivity at all spatial frequencies from 3 to 18 cycles/degree (*p* < 0.01 for 3 cycles/degree, *p* < 0.01 for 6 cycles/degree, *p* < 0.0005 for 12 cycles/degree, *p* < 0.05 for 18 cycles/degree). Values are expressed as the mean ± standard deviation. * *p* < 0.05, ** *p* < 0.01, *** *p* < 0.0005

Treatment resulted in significant increases in contrast sensitivity at all spatial frequencies from 3 to 18 cycles/degree (*p* < 0.01 for 3, *p* < 0.01 for 6, *p* < 0.0005 for 12, *p* < 0.05 for 18 cycles/degree; Figure 1).

### 3.3. Correlations between Changes in Tear Meniscus and Changes in AULCSF

The changes in AULCSF did not correlate with the changes in TMH (r = 0.009, *p* = 0.95) or TMA (r = 0.109, *p* = 0.41). The changes in log contrast sensitivity at each frequency were correlated with the changes in neither TMH (3 cycles/degree: r = −0.108, *p* = 0.41; 6 cycles/degree; r = 0.108, *p* = 0.42; 12 cycles/degree; r = −0.014, *p* = 0.91, 18 cycles/degree; r = −0.001, *p* = 0.99) nor TMA (3 cycles/degree: r = 0.026, *p* = 0.84; 6 cycles/degree: r = 0.082, *p* = 0.54; 12 cycles/degree: r = 0.114, *p* = 0.39; 18 cycles/degree: r = 0.016, *p* = 0.91).

### 3.4. Changes and Comparison of AULCSF in Eyes with NLDO Only and Those with the Involvement of Proximal Obstruction before and after LPI

**Figure 2:**
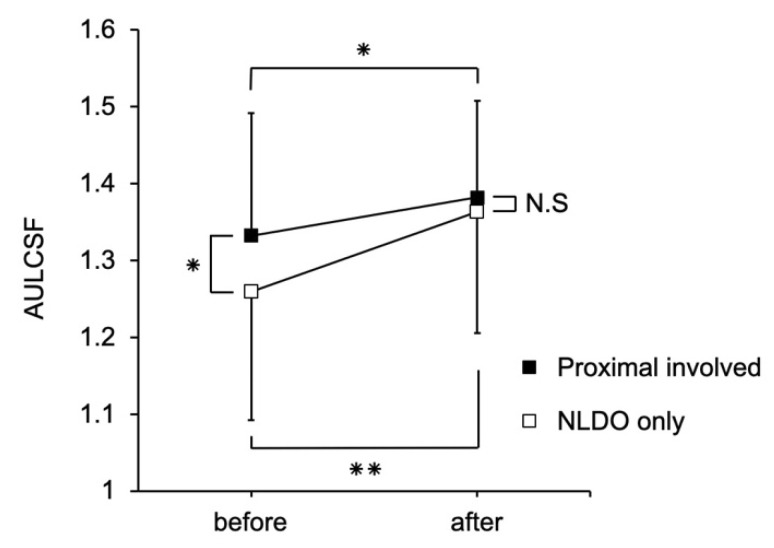
Changes and comparison of AULCSF in eyes with NLDO only and those with the involvement of proximal obstruction before and after LPI. The AULCSF improved significantly after LPI in both groups (NLDO only group, *p* < 0.001; proximal involvement group, *p* < 0.05). Before LPI, there was a significant difference in AULCSF between the two groups (*p* < 0.05); however, the difference became insignificant after LPI (*p* = 0.32). Values are expressed as the mean ± standard deviation. LPI, lacrimal passage obstruction; AULCSF, area under the log contrast sensitivity function; NLDO, nasolacrimal duct obstruction. * *p* < 0.05, ** *p* < 0.001

Figure 2 shows changes in AULCSF in eyes with NLDO only and those with the involvement of proximal obstruction. The AULCSF significantly improved after LPI in both the groups (NLDO only group: 1.26 ± 0.17 to 1.36 ± 0.16, *p* < 0.001; proximal involvement group: 1.33 ± 0.16 to 1.38 ± 0.13, *p* < 0.05). Before LPI, there was a significant difference in AULCSF between the two groups (*p* < 0.05); however, the difference became insignificant after LPI (*p* = 0.32).

There were no significant differences in TMH or TMA between the two groups before and after LPI (TMH before LPI: *p* = 0.11, TMH after LPI: *p* = 0.24, TMA before LPI: *p* = 0.37, TMA after LPI: *p* = 0.22).

## 4. Discussion

Contrast sensitivity tests are more sensitive for investigating the quality of vision than are standard visual acuity tests, which capture high spatial frequency channels well but do not necessarily predict vision at middle and lower frequencies [22]. Therefore, contrast sensitivity tests are useful for evaluating the quality of vision in eyes without or with slight decline in visual acuity. For example, dry eyes or eyes that underwent LASIK intervention show deterioration of contrast sensitivity, while their visual acuity remains unaffected [23,24]. In line with these findings, we previously reported a reduction in contrast sensitivity in eyes with epiphora caused by lacrimal passage obstruction, in which conventional visual acuity is maintained [1]. A contrast sensitivity test is also useful for evaluating the quality of vision before and after treatment in various anterior segment disease of the eyes, such as dry eye [25,26], ptosis and dermatochalasis [27], conjunctivochalasis [28], and cataract [29], as well as posterior segment eye diseases such as retinal detachment [30], epiretinal membrane [31], and posterior vitreous detachment [32], among others [33]. To the best of our knowledge, this is the first study to report improvement in the contrast sensitivity of the eyes after LPI for epiphora owing to lacrimal passage obstruction.

Regarding dry eyes—another common disease characterized by abnormalities in the tear film on the ocular surface—Koh et al. reported that AULCSF of dry eyes decreased to 1.24 ± 0.16, while that of normal eyes was 1.35 ± 0.11 [23]. Asano et al. revealed that eye drop treatment with diquafosol ophthalmic solution improves AULCSF from 1.26 ± 0.12 to 1.35 ± 0.14 in patients with SCL-related dry eyes [34]. In our study, LPI improved AULCSF from 1.29 ± 0.16 to 1.37 ± 0.14, which was comparable to the findings of the two above-mentioned reports, i.e., treatment for lacrimal passage obstruction with treatment for dry eye brings the same level of improvement in contrast sensitivity.

In this study, we found that AULCSF before LPI was significantly worse in the NLDO only group than in the proximal involvement group. When we performed lacrimal passage system irrigation, reflux fluid often contained mucus and/or pus in the eyes with NLDO alone but not in those with proximal involvement. Differences in the turbidity of tear meniscus between eyes with NLDO alone and those with proximal involvement may affect the difference in contrast sensitivity. Hiraoka et al. reported that increased light scattering after instillation of brinzolamide causes deterioration of contrast sensitivity [35]. In patients with NLDO, it is possible that light scattering may increase because of excessive retention of proteins in tear film that is not excreted into the nasal fossa, while this is less common in the eyes with proximal involvement. In contrast, tear meniscus volume did not seem to directly affect contrast sensitivity because there were no significant differences in TMH or TMA between the NLDO only and proximal involvement groups. The AULCSF improved after LPI in both the groups, and there was no difference in AULCSF between both groups after LPI. This may suggest that LPI leads to normalization of tear content, resulting in the improvement of contrast sensitivity to the same level in both cases. Although it is not volume-dependent, excessive tear meniscus volume can lead to instability of the tear film on the corneal surface, which can affect contrast sensitivity in eyes with lacrimal passage obstruction.

Improvement in AULCSF was not correlated with changes in tear meniscus parameters in this study. Our previous study including unilateral lacrimal passage obstruction cases also revealed no correlation between AULCSF and tear meniscus parameters [1]. Similarly, Koh et al. reported that tear meniscus parameters were not correlated to the quality of vision or optical quality in patients with epiphora owing to nasolacrimal passage obstruction [2]. From these results, tear meniscus volume does not seem to affect visual quality in patients with lacrimal passage obstruction. One possible explanation would be that the tear film varies and has different phases with blinking; therefore, the condition of tear meniscus is not constant between OCT measurement of the tear meniscus and the visual quality tests.

This study had some limitations. Contrast sensitivity and the other parameters were not recorded with the stent in place, since postoperative measurements were performed one month after removal of the stent. As many patients are aware of improvement in their visual performance soon after LPI, with the stent in place, contrast sensitivity and the other parameters should also be investigated at that stage, to precisely describe the effect of LPI on vision. Another limitation was the rather short follow-up period in the study. Patency decreases with follow-up, and long-term results of LPI are not always satisfactory [7,36]. Recurrence of stenosis and obstruction of the lacrimal passage during longer follow-up may affect the quality of vision. Further investigations are needed to clarify the long-term effects of LPI on visual function including contrast sensitivity.

## 5. Conclusions

In conclusion, contrast sensitivity significantly improved after LPI in eyes with epiphora owing to lacrimal passage obstruction. Contrast sensitivity measurement before and after LPI might aid in our understanding of the effectiveness of treatment on the recovery of visual function in eyes that underwent LPI.

## Figures and Tables

**Table 1:** Type of obstruction as determined using dacryoendoscopy.

Underlying Disease	Eyes (*N*)
Nasolacrimal duct obstruction only	32
Proximal involved	26
Common canalicular obstruction combined with nasolacrimal duct obstruction	14
Common canalicular obstruction	10
Upper and/or lower punctal obstruction	2

**Table 2:** Comparison between measured parameters before and after lacrimal passage intubation.

Parameters	Before LPI	After LPI	*p* Value
BCVA (logMAR)	−0.10 ± 0.06 (−0.08, −0.18 to −0.08)	−0.10 ± 0.05 (−0.08, −0.18 to −0.08)	^a^ 0.61
AULCSF	1.29 ± 0.17 (1.31, 1.17 to 1.44)	1.37 ± 0.14 (1.39, 1.28 to 1.49)	^b^ <0.0001
Tear meniscus height (mm)	0.46 ± 0.20 (0.46, 0.31 to 0.58)	0.34 ± 0.11 (0.33, 0.24 to 0.42)	^b^ <0.005
Tear meniscus area (mm^2^)	0.09 ± 0.07 (0.07, 0.04 to 0.11)	0.04 ± 0.03 (0.04, 0.03 to 0.06)	^a^ <0.005

Data are presented as the mean ± standard deviation (median, interquartile range). The ^a^ *p* value, evaluated using the Wilcoxon signed-rank test. The ^b^ *p* value, evaluated using a paired t-test. LPI, lacrimal passage obstruction; BCVA, best-corrected visual acuity; AULCSF, area under the log contrast sensitivity function.
